# Suppression of COVID-19 death incidence on open west coasts in the USA

**DOI:** 10.1038/s41598-025-12972-x

**Published:** 2025-08-05

**Authors:** Karin Ebert, Jani Turunen, Renate Houts, Sergio Noce, Siddartha Aradhya

**Affiliations:** 1 Department of Environment, Development and Sustainability Studies, School of Natural Sciences, Environment and Technology, Södertörn University, Stockholm, Sweden; 2 Department of Sociology, Stockholm University, Stockholm, Sweden; 3 Department of Social Work, School of Social Sciences, Södertörn University, Stockholm, Sweden; 4 Department of Psychology and Neuroscience, Duke University, Durham, NC USA; 5 CMCC Foundation - Euro-Mediterranean Center on Climate Change, Viterbo, Italy

**Keywords:** COVID-19 death incidence, Continentality, Spatial pattern, Oceanic influence, GIS, Infectious diseases, Environmental impact, Environmental impact

## Abstract

The drivers behind the large-scale patterns of COVID-19 infections are largely unknown. Earlier studies have shown a connection between continentality, a measure for oceanic air influence over land, where lowest continentality implies highest oceanic influence, and COVID-19. In Europe, open west coasts with lowest continentality had the lowest COVID-19 incidence. We test if this applies to the US. We use a combination of geographical information systems and statistics, and data for every US county, to assess the connection between the COVID-19 death incidence and continentality. We normalize for known factors that influence COVID-19 local scale death incidence, namely the socio-economic status, population aged over 65, and the index of urbanization (crowding). We find that open west-coasts in the US, where continentality index values are low, had the lowest COVID-19 death incidence, rising non-linearly with rising continentality values, with highest death incidence in areas with the highest continentality, in north-central USA. The influence of oceanic air was associated with lower COVID-19 death incidence on the west coast of the US. These findings suggest that oceanic influence may be an important environmental determinant of spatial variations in COVID-19 death incidence and provide a contribution to studies on the relationship between oceans and health.

**Supplementary Information:**

The online version contains supplementary material available at 10.1038/s41598-025-12972-x.

## Introduction

Infections caused by a number of respiratory viruses such as influenza and respiratory syncytial virus are known to have a partially predictable seasonal occurrence and intensity that differs geographically across the Earth’s latitudes and climate zones^1–4^. Despite decades of research, the drivers behind these patterns are still not fully understood^5^. The COVID-19 pandemic, caused by the respiratory virus SARS-CoV-2, showed similar seasonal patterns^6^. The abundance of collected data during the pandemic^7^ lead to a vast number of studies, trying to couple, amongst others, weather and human behavior to virus occurrence. However, many unsolved questions about factors triggering virus death incidence patterns remain^4^, both on local and especially regional scale. In our study we investigate the regional scale.

We approach the problem from a spatial perspective, to establish if discernible patterns of higher and lower COVID-19 death incidences occurred in the USA, and to find possible environmental triggers for such a pattern. We use the death incidence, even though possibly underreported^8^, a more rubust measure than the case incidence, that can vary even more, depending on, for example, test numbers^9,10^. The detailed data that was collected daily for each county in the USA during the pandemic gives the opportunity to analyze spatial patterns of infection occurrence and intensity for these small spatial entities (> 3000 counties). Data on virus patterns are often analyzed country-wise, and therefore, the US is often treated as one entity in seasonality studies of respiratory viruses^2^. The US is consequently treated and described as one area with several COVID-19 peaks during all seasons, as for example seen in the Johns Hopkins data^7^ for the entire country combined. However, the US encompasses a wide range of longitudes and latitudes, population density, age structure and climate zones. We examine the curve by analyzing the COVID-19 pattern at the county level across the US. To date, however, a detailed spatial analysis assessing this relationship in the context of the diverse climatic and demographic landscape of the United States has not been performed.

Earlier studies for European counties have shown a correlation between open west coasts and lower COVID-19 incidence, and rising case incidence with rising distance to the coast, consequently, a correlation between continentality and oceanic influence on the COVID-19 pattern^10,11^. A study on COVID-19 mortality for 569 European regions found the same east–west gradient^12^. Europe is located in the westwind-zone of the northern hemisphere, with predominantly westerly winds that carry sea air on the continent, causing a mild climate with low continentality—low temperature differences throughout the year – in the west coast near zones. The westwind zone is a part of the global wind system. It stretches across the northern hemisphere at roughly 30° to 60° N. Continentality is intended as a climatic characteristic describing how much a place’s climate is influenced by its distance from oceans or large bodies of water. The transport of mild oceanic air over land leads to low continentality, while its absence results in higher temperature variablity. Areas with high continentality tend to experience wider temperature extremes, both seasonally and daily, due to the absence of the moderating influence of water. Large annual ranges in air temperature result in higher index values and consequently indicate a more coninental climate, whereas the smallest differences can be observed in the most oceanic climate conditions. Continentality can therefore be considered a useful proxy for oceanic influence^13^, as it reflects the degree to which maritime air masses shape local climate variablity. In this context, maritime (low-continentality) climates—, typically found closer to large bodies of water and located in prevaling in wind zones from sea to land—are characterized by narrower temperature ranges^14^. This is a general (global) rule, some areas (like Alaska) are exceptions for several reasons: (1) regions closer to the poles, such as Alaska, experience dramatic changes in daylight hours throughout the year, leading to hot summers and very cold winters^15^; (2) the peculiar topography of Alaska, particularly in interior regions acts as a barrier to oceanic air masses^14^; (3) the sub-arctic climate features short summers and long, harsh winters^16^. These aspects contribute to increasing the continentality values of this peculiar region. Mainland USA comprises a huge landmass in the northern hemisphere, mainly in the same zone with predominant westerly winds or “westerlies” (with exception of Alaska, that is located north of 60° N, in the zone of the polar easterlies, and the island of Hawaii, in the Pacific Ocean south of 30° N, in the zone of the northeast trade winds). The vastness of the landmass, with a ~ 4000 km west-to-east-extension, and > 1000 km of west coast, allows us to test whether the influence of continentality holds true even for this area. Westerlies are one of the key-factors underlining the differences in continentality values between the two coasts, in the West Coast these winds move ocean air from the Pacific Ocean (moderating climate), on the East Coast the prevailing winds carry continental air, resulting in more extreme temperatures^17^. Another important aspect is that the air masses that blow from the Pacific are greatly slowed down (if not blocked) by imposing mountain barriers (Sierra Nevada)^14^. Finally, the cold Labrador current that persists on the East Coast causes a notable impact on the northern section (except Florida) especially in the winter season^15^.

Therefore, we aim to investigate the relation of the COVID-19 death incidence pattern in the United States to continentality, i.e. the death incidence in relation to oceanic influence. We normalize the data against the most known factors for virus impact, i.e. the degree of urbanization^18^ (crowding), the share of over 65-year-olds in the population^19^, and the socio-economic status (SES), which had noticeable effects on the COVID-19 death incidence^20,21^. We analyze the total death incidence for each county during the period from January 2020 to the end of March 2022.

## Materials and methods

We use a combination of Geographical Information Systems (GIS) in ArcGIS Pro 3.1 and statistics in Stata 18 and R. The combination of GIS and statistics enables us to analyze correlations between environmental parameters and COVID-19 death incidence per 100 000 inhabitants for each county, and to present the parameters and resulting correlations spatially on maps.

### Data description

We use COVID-19 death incidence data^22^, the continentality index^23^, demographic data with percentage of ages 65 and older^19^, the index of urbanization, the SES index, and a county boundary dataset for all counties of the US^24^, and integrate these data in ArcGIS Pro 3.1 for preparation and visualization, and for statistical analysis.

**Fig. 1:**
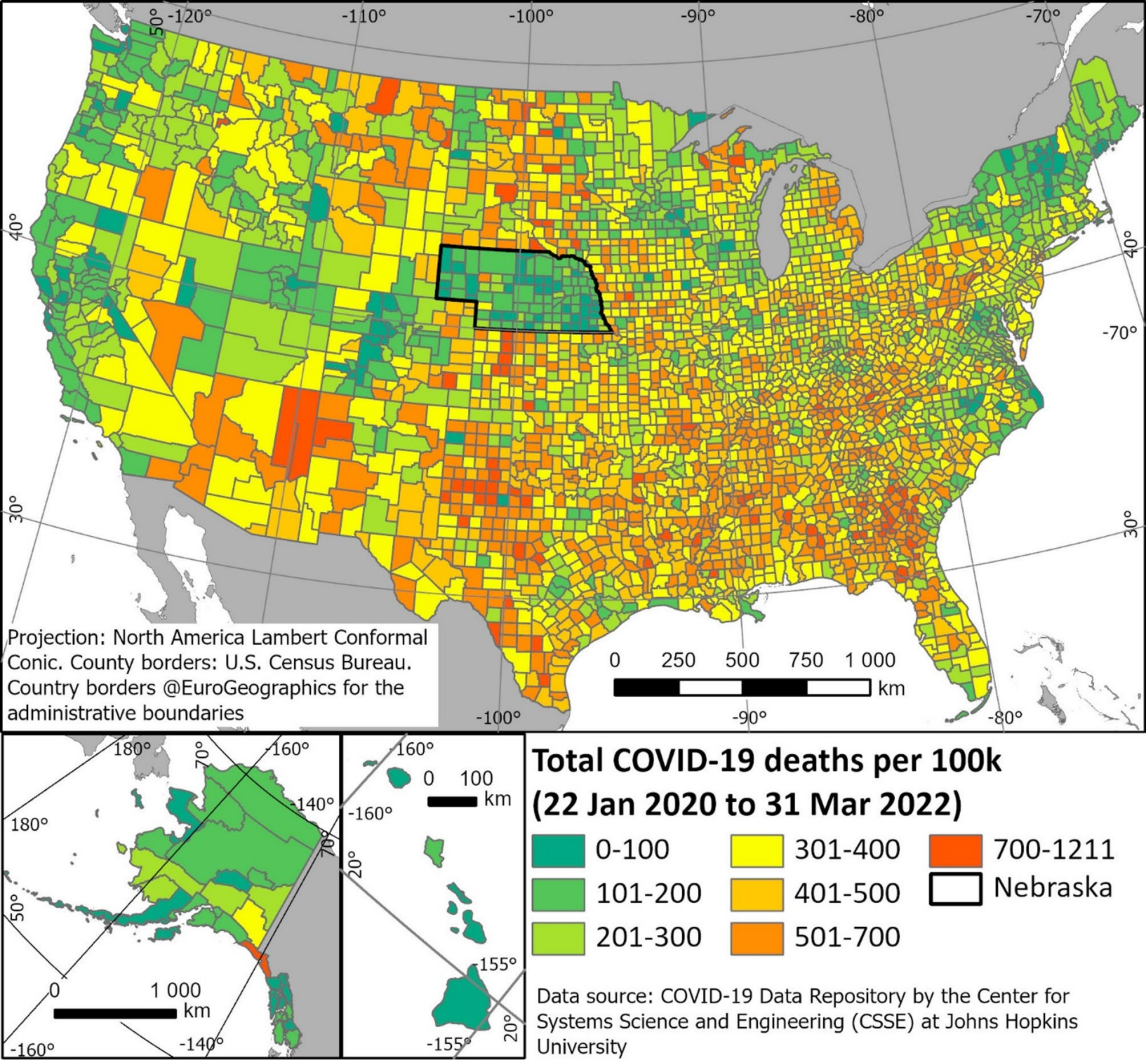
Total (accumulated) county-wise death incidence for COVID-19 per 100 000 inhabitants, from Jan 22, 2020 to March 31, 2022. Data from JHU CSSE COVID-19 Data, https://github.com/CSSEGISandData/COVID-19^22^. The State of Nebraska, with a deviating death incidence to neighboring states, is indicated with thicker borders.

Johns Hopkins *cumulative total county-level COVID-19 deaths from Jan 22, 2020 through Mar 31, 2022* were obtained from the COVID-19 Data Repository by the Center for Systems Science and Engineering (CSSE) at Johns Hopkins University^22^. The data display the 7-day average death numbers. Totals were calculated from the Johns-Hopkins time-series data and converted to death incidence/100 K. For visualization, the resulting table was joined with the attribute table for the administrative borders^24^ of all counties (Fig. 1).

In our calculations we used *Continentality index data* from the CMCC-BioClimInd data set^23^. This dataset provides data of 35 bioclimatic indicators from historical and future climate simulations. The historical data (1960–1999) employed in our analyses have a spatial resolution of 0.5° and are based on WATCH reanalyses. In this dataset the continentality index is calculated in its simplified form^23^, this differs from the one developed by Driscoll and Yee Fong (1992) and is calculated as the difference (in °C) between the mean temperature of warmest and of the coldest months of the year. The simplified continentality index used here differs from Driscoll and Yee Fong’s formula by measuring only the difference between the mean temperatures of the warmest and coldest months, without additional weighting factors. This straightforward method relies on monthly temperature data, ensuring robustness and consistency across regions. By directly capturing the seasonal temperature amplitude, it provides a clear and intuitive measure of continental influence. Its simplicity facilitates large-scale spatial comparisons and reduces calculation errors. Therefore, it is well suited for broad regional analyses where data comparability has a strong weight.

The *percentage of over 65 year olds* in the population, obtained from the U.S. Census Bureau’s American Community Survey from 2019^19^ is a measure that gives the share of the over 65 year olds in the population for each county. We adjusted for the percentage of over 65 year olds as older people were overrepresented in the COVID-19 death statistics^9^ and also tend to live in coastal areas, like Florida^19^, where the continentality index is low, to a greater extent. Emerging evidence also suggests that depressive symptoms in middle-aged and elderly individuals significantly elevate the risk of cognitive decline and dementia, which may indirectly exacerbate COVID-19 related mortality risks through compounded health vulnerabilities^25^.

The *degree of urbanization* provides the relationship between the population living in urban (and rural) areas and the total population of the municipality^26^, with other words, population accumulations, or areas of crowding, are identified^27^ (Supplementary file 1).

The *SES index* combines the five factors poverty, unemployment, the housing cost burden, no high school diploma and no health insurance^28^ (Supplementary file 2).

### GIS visualization and data preparation

**Fig. 2:**
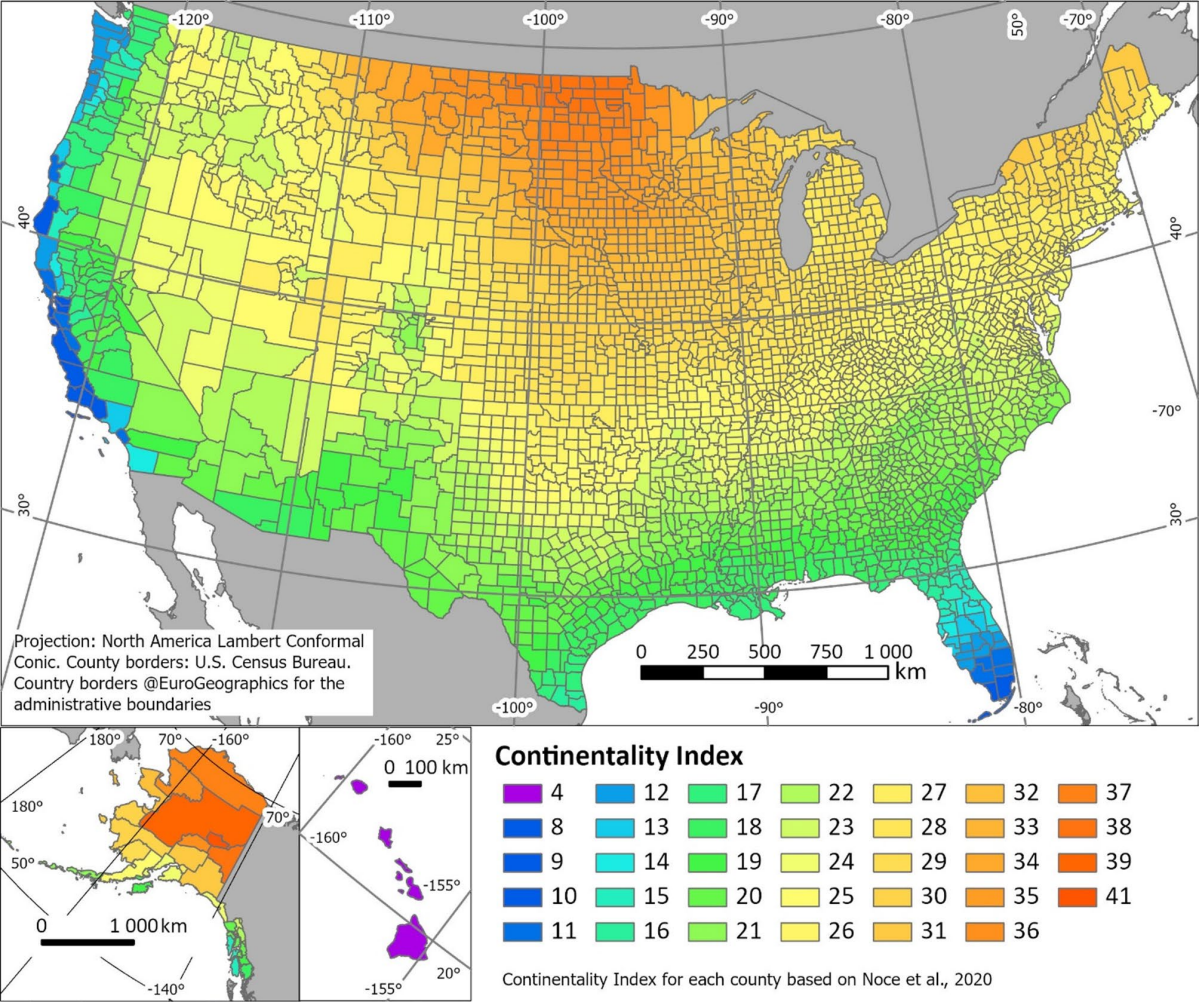
Average continentality index for each US county (modified from Noce et al.^23^).

The average continentality value for each county was obtained by spatial join of the county administrative borders file with the continentality raster file^23^ (Fig. 2).

The spatial distribution of the average index of urbanization value for each county was obtained in the same way, from the raster provided by Eurostat^29^.

**Fig. 3:**
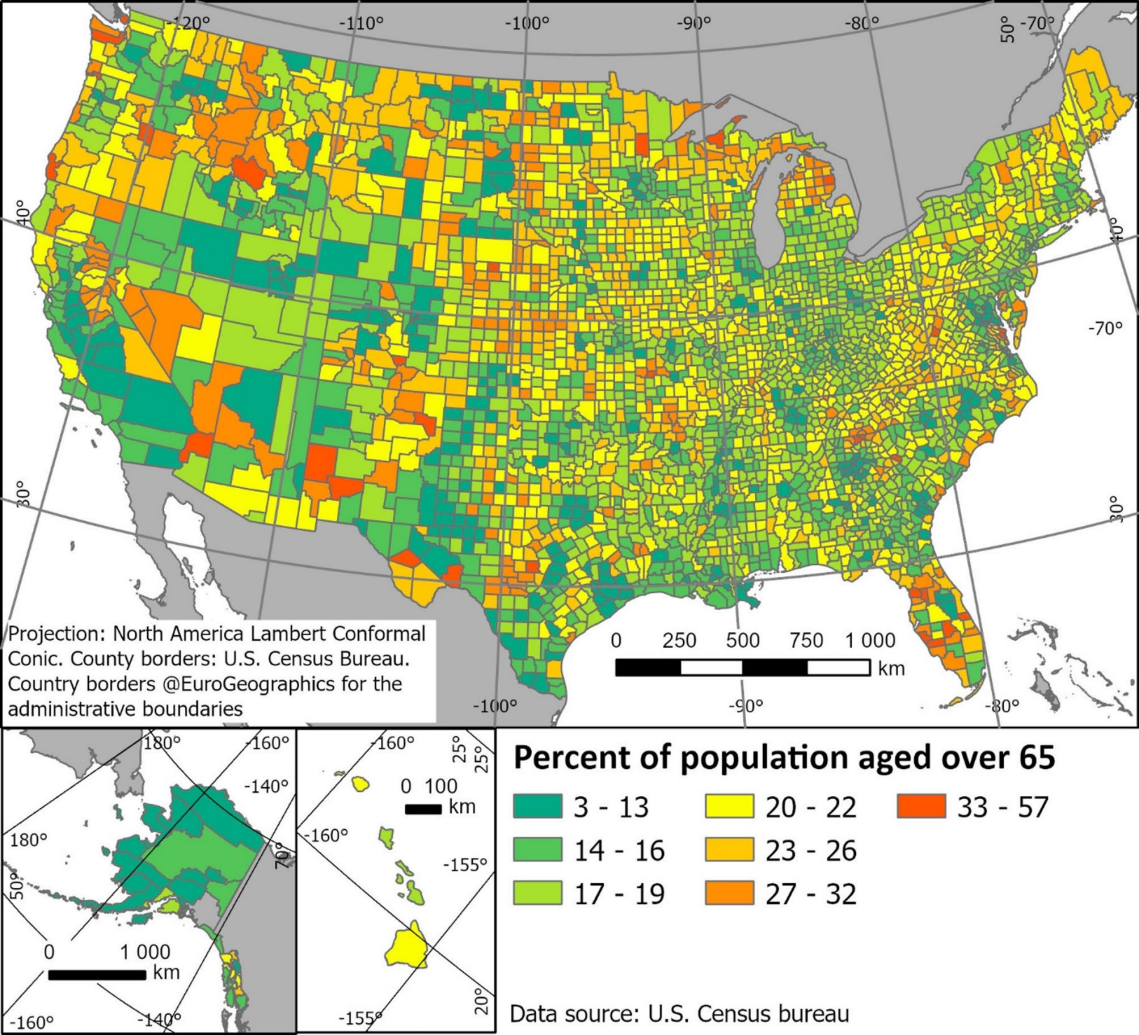
Percent of population 65 years and older, from U.S. Census bureau^19^. We normalize against the share of over 65 year olds in the population, because elderly were over proportionally affected by COVID-19 caused deaths^20,21^.

The percentage of the population aged over 65y for each country was obtained in tabular form from the US Census Bureau and joined to the administrative border attribute table for visualization (Fig. 3).

The SES for each country was obtained in tabular form from the center of disease control (CDC)^28^ (Supplementary file 2).

The resulting table of all joins, containing COVID-19 death incidence per 100.000 citizens, the average continentality value, the average index of urbanization value, the percentage of over 65-year-olds of total population and the SES value for each country was downloaded and used for the statistical analyses. The resulting coefficient was joined back to the attribute table in ArcGIS for visualization.

### Statistical analysis

**Fig. 4:**
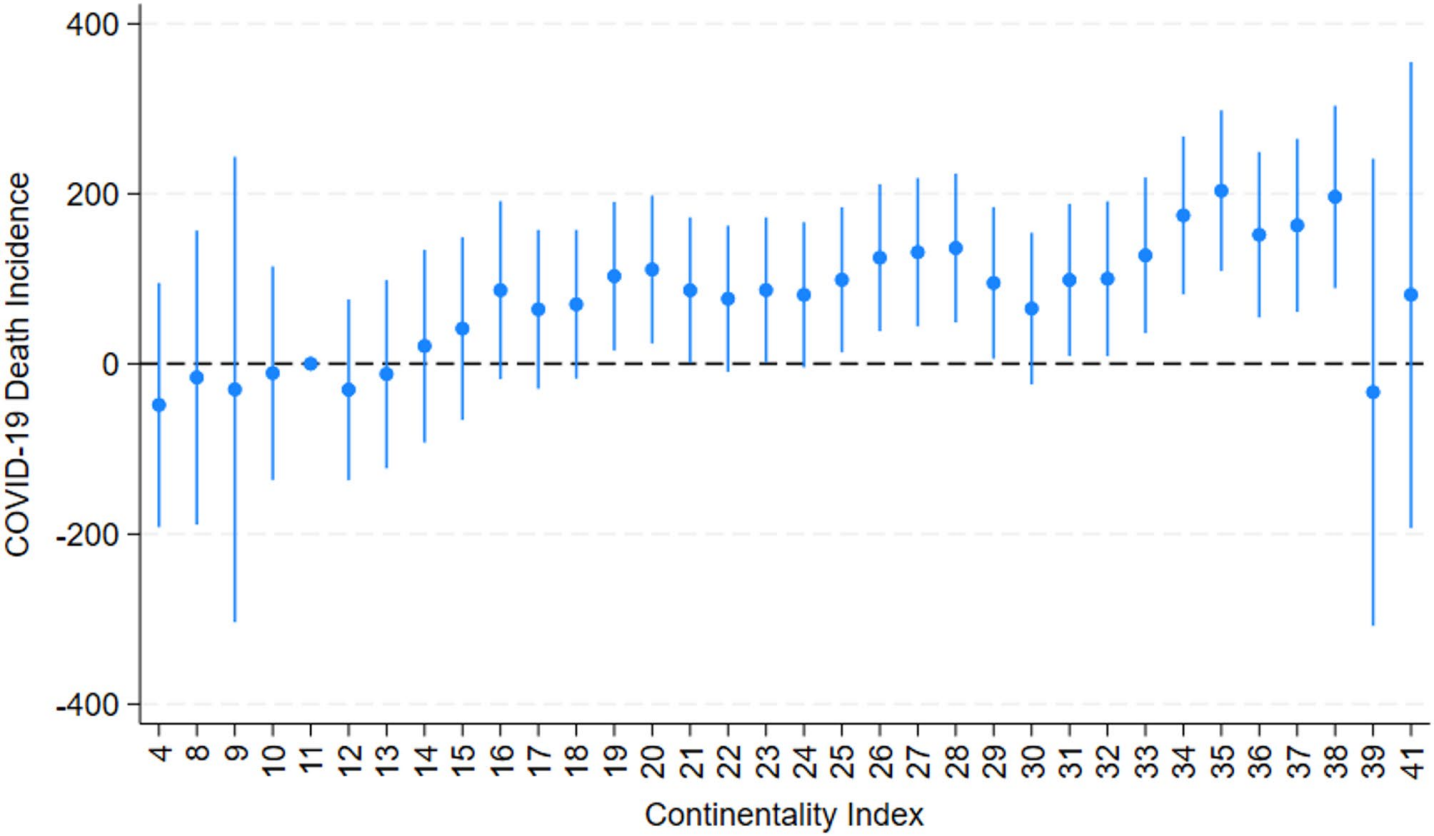
Coefficient plot of Continentality index and COVID-19 death incidence with Continentality index 11 as the reference category. Dots represent coefficient estimates, and the bars reflect 95% confidence intervals. Continentality values 39 and 41, that deviate from the trend, only occur in one county each and cannot be regarded as statistically robust. See visualization of coefficient across the US in Fig. 5.

**Fig. 5:**
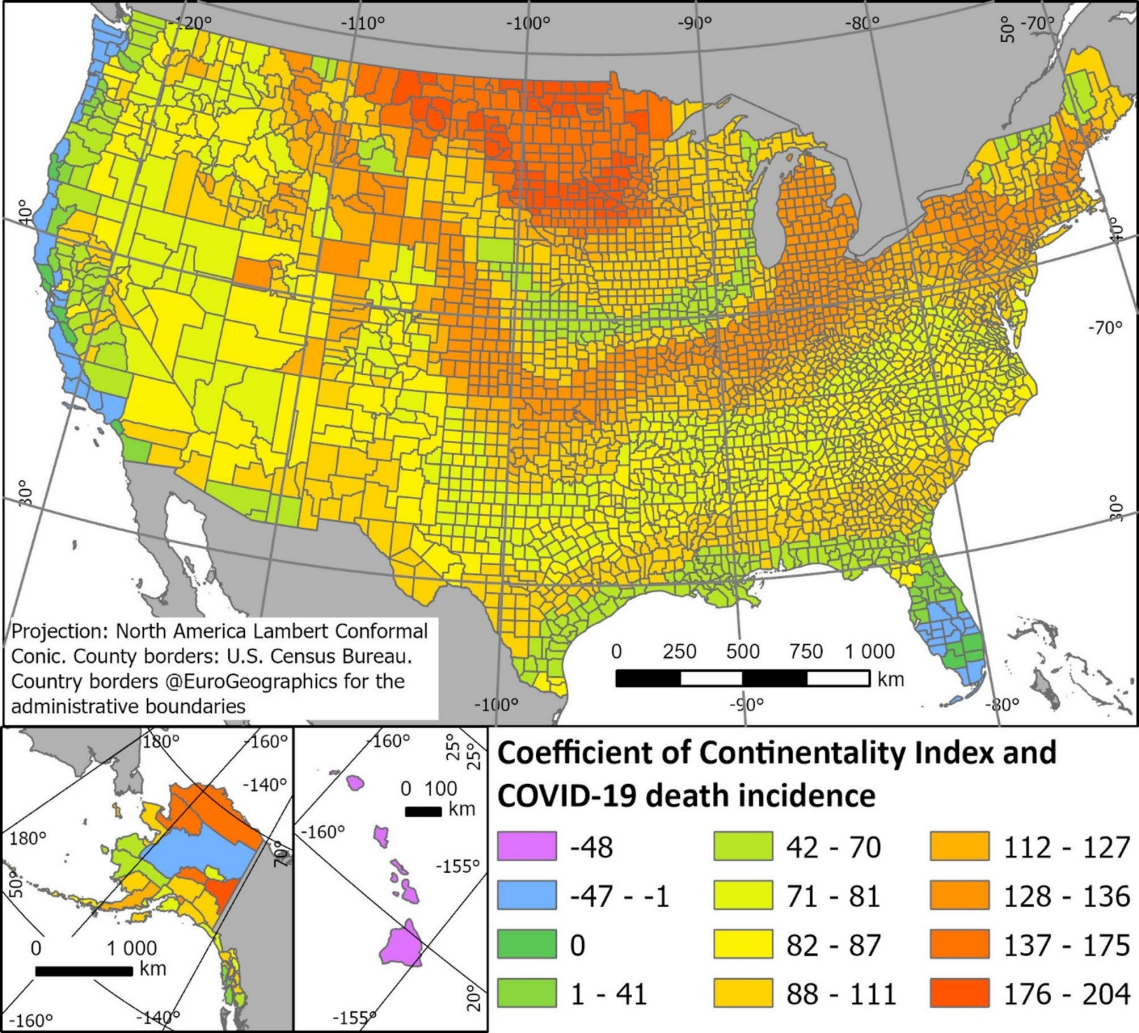
Spatial visualization of Coefficient per US county of Continentality index and COVID-19 death incidence. The coefficient shows how differences in the continentality index are associated with changes in COVID-19 death incidence within the same U.S. Census region, after adjusting for age structure, SES, and crowding. The lower the coefficient, the lower the relative probability of dying in COVID-19.

We fit linear regression models with regional fixed effects in order to estimate the relationship between continentality index and COVID-19 death incidence (Fig. 4) and visualized the values in Fig. 5 (Table: Supplementary file 3).

To estimate the relationship between the continentality index and COVID-19 death incidence, we specify a fixed effects regression model that accounts for the nested data structure, where counties are grouped within broader geographic areas. The dependent variable, ${y}_{ic}$, represents the COVID-19 death incidence measured at the county level *c*, within area *i*. The main explanatory variable is the continentality index, also measured at the county level. In addition, we include a vector of control variables ${X}_{ic}$, which capture other observable county-level characteristics that may be associated with the outcome.

The regression model is specified as follows:

$${y}_{ic}={\beta }_{1}{Continentality}_{ic}+{X}_{ic}^{\prime}{\beta }_{2}+{a}_{i}+{\varepsilon }_{ic}$$

In this equation, ${Continentality}_{ic}$ is the variable of interest, ${X}_{ic}$ is a vector of county-level controls, and ${\beta }_{2}$ is a corresponding vector of coefficients. The term ${a}_{i}$ denotes area fixed effects, which absorb all shared unobserved heterogeneity at the area level that may confound the relationship between continentality and COVID-19 death incidence. The error term ${\varepsilon }_{ic}$ captures unobserved variation in the outcome at the county level.

The inclusion of area fixed effects ${a}_{i}$ controls for any persistent characteristics that are common to all counties within a given area—such as regional institutions, historical development patterns, or baseline policy environments—thereby ensuring that the estimated effect of the continental index is identified solely from variation within areas. That is, the fixed effects remove between-area variation from the estimation, allowing the model to compare counties with different values of the continental index but within the same broader area.

The coefficient of interest, ${\beta }_{1}$, thus captures the average within-area association between the continental index and the outcome. It reflects how differences in the continental index across counties within the same area are associated with differences in the outcome, conditional on the included control variables and unobserved area-level characteristics. In other words, ${\beta }_{1}$ estimates how much the outcome changes, on average, when the continental index differs between two counties in the same area, all else being equal.

To account for potential spatial or institutional correlation in the error structure, we cluster standard errors at the area level. This corrects for arbitrary forms of heteroskedasticity and autocorrelation within areas and ensures valid statistical inference in the presence of within-area dependencies.

In addition, we assessed multicollinearity among covariates using the Variance Inflation Factor (VIF) and examined the normality of residuals. Neither diagnostic indicated any concerns.

The inclusion of regional fixed effects allows us to adjust for all factors shared within a region; as such the parameters are estimated exploiting within region variation. This approach is ideal since we adjust for all unobserved factors that are fixed within each region (as defined by the US Census Bureau as South, Midwest, West, and Northeast), such as labor market differences and regional variation in political ideologies. We include a parsimonious set of confounders such as degree of urbanization (crowding), SES index, and the share of the population over 65 years of age in each county.

All analyses used continentality index 11 as the reference category (arbitrary, as the reference category is needed but has no influence on the outcome), so all coefficients in the continentality index correspond to deviations from counties that experience an 11-degree difference between the highest and lowest temperatures in a year. We also conducted robustness checks where we exclude the state of Nebraska, as there appear to be inconsistencies in reporting of COVID-19 death incidence. Nebraska has a noticeably lower death incidence than the surrounding states of Kansas and South Dakota, and in line with California, a state with one of the lowest death incidences, as shown also in other diagrams of the Johns Hopkins data for the states of the US, for example on the webpage 91-divoc.

We did a robustness check and calculated the coefficient by omitting Nebraska. The changes were marginal; for the results presented in this paper, we used the data as they are, including Nebraska.

We chose to normalize our data against the most known factors for the increase in COVID-19 death incidence: the socio-economic status, the age group 65 and older, and crowding. These are all factors within the Social Vulnerability Index (SVI)^28^. We chose against normalizing for the entire SVI, as the SVI also includes measures that are not known to influence COVID-19 death incidence, such as the population aged 17 and younger. However, we tested normalizing against the entire SVI, and the statistical differences in the spatial pattern were not significant (Supplementary file 4), confirming a lower COVID-19 death incidence in areas with lower continentality and a non-linear rise, with highest death incidence in the areas with highest continentality.

We did not normalize our data against other parameters that likely had local impacts on COVID-19 death incidence, such as distancing rules and vaccinations. These are parameters that varied in space and time across the US states during the years of the pandemic^30^, and suitable spatial data for the regional-scale perspective of our study does not exist. However, in normalizing our data against the SES, we covered some of these parameters, as socio-economic status, which includes access to information, access to health care, and housing conditions, shows a positive correlation with follow stay-at-home orders^31^ and getting vaccinated^32^.

## Results

The pattern of the total COVID-19 death incidence per 100,000 citizens, from 22 January 2020 to 31 March 2022, the main phase of the pandemic (Fig. 1), shows a lower death incidence along the west coast and the center of the Rocky Mountains, and higher death incidence numbers east of the Rocky Mountains, and especially in the southern States. An exception on the map is Nebraska that shows an unproportional low death incidence, compared to all neighboring States. We could not find a reason for this and treated it as is.

The continentality index across the US shows a huge span of between 4 and 41 degrees Celsius difference throughout the year (Fig. 2). The lowest value is reserved for Hawaii with doubtless the highest oceanic influence across the islands. Low values with yearly variations under 10 degrees Celsius are found at the Californian coast and the southern tip of Florida. Continentality values rise from both these areas inland and northwards, with the highest yearly temperature differences in North Dakota and inland Alaska. As a general trend, the index is confirmed to increase with the distance from the coast, but this is not observed in the northeastern portion of the United States.

Higher percentages of population aged over 65 are distributed unevenly across the country (Fig. 3) with certain regions displaying a higher share of retirees (For example, Florida).

The Coefficient plot of Continentality index and COVID-19 death incidence (Fig. 4) shows lowest coefficient values (i.e. lowest probability to die in COVID-19) for the lowest continentality values. The coefficient shows a non-linear rise, with the highest coefficient for the highest continentality values up to 38 degrees Celsius temperature variation per year.

The two highest continentality values 39 and 41 deviate from this trend, with a clearly lower death incidence. However, we cannot regard these as statistically robust, because both values only occur in one county each (39 in Yukon Koyukuk, Alaska and 41 in the neighboring county Fairbanks North Start, Alaska). The next highest value, 38, occurs in 17 counties (of 3204 counties in total). We mapped the coefficient (Fig. 5) to spatially show the relative probability of COVID-19 death incidence between January 2020 and March 2022 per county and continentality zone. The entire west-coast towards the Pacific, as well as Florida and Hawaii, show low coefficients and thereby the lowest probability for death in COVID-19. Coefficient values are rising following the Continentality index inland and northwards, not entirely linearly, but with the highest coefficient values in the area with the highest Continentality index, thereby lowest oceanic influence and highest temperature differences between minimum and maximum temperature per year, in North Dakota and north-eastern Montana.

## Discussion

Our study corroborates previous findings that indicate lower COVID-19 incidence in the open west coasts, within the northern Hemisphere west-wind zone and a progressive increase with rising continentality^10,11^. This suggests that continentality, a measure of the variation in temperature influenced by proximity to large bodies of water, plays a role in the spatial distribution of COVID-19 death incidence. While the exact mechanisms behind this association require further investigation, our results show a clear relationship between oceanic influence and a suppression of COVID-19 death incidence from January 2020 to March 2022.

Coastal areas have long been associated with health benefits, historically serving as sites for recuperation and wellness activities^33^, and have long been used as locations for recuperation, often associated with activities like exercise, exposure to sunlight, and relaxation^43^ Our findings underscore this, showing the strongest reduction in COVID-19 incidence along the open west coast, in the northern hemisphere’s predominant west-wind zone. While behavioral factors such as healthier lifestyles in coastal regions may partly explain this trend, the data points towards an additional, more direct influence of the ocean on public health.

Low continentality values serve as a proxy for higher oceanic influence, with mild sea air having a moderating effect on temperature variability. Recent studies in an emerging area of epidemiology support the idea that sea air, specifically marine aerosols, may have a positive impact on lung health^34–36^. Data from Belgium have shown that populations living less than 5km from the coast reported a general better health status than populations further inland^37^. Studies have suggested immune protective effects of marine aerosol, in particular marine bacteria and endotoxins^38^. Marine aerosols comprise potentially beneficial components, such as biogenic surfactant^36^ and salts^39^, which may contribute to better respiratory health and reduce the severity of respiratory infections, including COVID-19^35,39^. Additionally, vitamin D deficiency has been shown to correlate with greater COVID-19 severity, and supplementation in deficient individuals may reduce mortality, suggesting environmental exposures and micronutrient availability may also influence regional outcomes^40^.

A potential future avenue of research could focus on the temporal development of the COVID-19 pattern in relation to the transport of sea air over land. By examining how fluctuations in sea air components correlate with changes in COVID-19 death incidence, we could better understand the role that oceanic influence plays in mitigating the virus’s effects.

In our analysis, we normalized for local factors known to affect COVID-19 death incidence, such as age demographics, socioeconomic status (SES), and population density. This allowed us to isolate the broader, spatial regional patterns of COVID-19 infection rates. The trend we observed—a marked increase in COVID-19 deaths moving inland from the (west) coast—persisted even after accounting for these variables. It is important to acknowledge, however, that numerous factors influenced the course of the pandemic, including vaccination rates and public health measures like social distancing, which varied widely across different states. Despite normalization for SES and age structure, unmeasured confounding from variations in public health policy and vaccine uptake likely influence observed spatial differences. While our SES index captures multiple aspects of social vulnerability, education level has also been shown to independently predict mortality risk and may interact with pandemic outcomes through pathways such as health literacy, occupational exposure, and adherence to public health recommendations^41^. Behavioral patterns such as increased digital device use during the pandemic may reflect regional variations in isolation practices and public health messaging, factors that can mediate infection outcomes across socio-demographic groups^42^. These local variations may contribute to the overall pattern, but our focus on regional trends provides a clearer view of how environmental factors, such as continentality, may be shaping COVID-19 patterns on a broader scale.

One notable exception is Nebraska, which exhibited a disproportionately low COVID-19 death incidence compared to neighboring states. We were unable to identify any clear confounding factors for this anomaly, which underscores the complexity of the pandemic and the many variables at play. Further research is required to fully understand the intricate interactions between geographic, environmental, and social factors that influence the spread and impact of SARS-CoV-2.

While introducing a new and innovative perspective on the study of this disease’s spread, our work carries several limitations. Studies linking the continentality index to COVID-19 incidence reveal associations but do not establish direct causality. Although factors such as index of urbanization and demographic structure are controlled for, potential confounders like mobility, public health policies, and data quality remain. The broad spatial and temporal scales may obscure important local variations, and the relatively coarse resolution of environmental data introduces bias by overlooking finer climatic differences. While the continentality index is useful, it does not account for other relevant variables. Additionally, epidemiological data may be incomplete or underreported, and indirect effects of climate on human behavior, which are difficult to quantify, are rarely included. Therefore, these findings should be interpreted cautiously and primarily at broad spatial scales, considering continentality as one of multiple factors influencing virus spread.

Summarizing, our study highlights the importance of oceanic influence, as measured by continentality, in shaping the geographical distribution of COVID-19 death incidence. Coastal regions with higher oceanic influence, such as the west coast, Florida, and Hawaii, exhibited lower mortality rates, while inland areas with higher continentality experienced higher death rates. Although behavioral, social, and policy factors undoubtedly played a role, our findings suggest that the physical environment, particularly the proximity to oceans and the corresponding climate effects, may be an important but underexplored factor in the global response to respiratory pandemics like COVID-19. Future studies should continue to explore this relationship, investigating not only the spatial patterns of disease spread but also the potential benefits of sea air components and other environmental factors in mitigating respiratory infections.

Our findings confirm that open US west coasts in the west wind zone of the northern hemisphere, where continentality index values are low and oceanic influence is high, showed the lowest COVID-19 death incidence. The results help us understand potential low- and high-risk zones of COVID-19 infection and death incidence and provide a puzzle piece for studies on the relationship between oceans and health.

## Electronic supplementary material

Below is the link to the electronic supplementary material.

Supplementary Material 1

## Data Availability

The data that support the findings are openly available in public repostitories. Administrative county borders US and US population numbers: Source U.S. Census bureau, link: https://hub.arcgis.com/datasets/esri::usa-counties/about. Administrative country borders, countries around US: @EuroGeographics for the administrative boundaries, link: https://gisco-services.ec.europa.eu/distribution/v2/. COVID-19 death numbers: Johns Hopkins Coronavirus resource center, link: https://github.com/CSSEGISandData/COVID-19. Aged over 65 per county: U.S. Census bureau, tabular data was sent to us upon request. SES: part of the SVI (Social Vulnerability Index), links for SVI explanation and download: https://www.atsdr.cdc.gov/placeandhealth/svi/index.html. https://www.atsdr.cdc.gov/placeandhealth/svi/data_documentation_download.html. Degree of urbanization: GHSL (Global Human Settlement Layer), link: https://data.jrc.ec.europa.eu/dataset?keyword=global%20map. See also supplementary files 1–4. The datasets used and/or analysed during the current study are available from the corresponding author on reasonable request.
