# Supplementary material for: Suppression of COVID-19 death incidence on open west coasts in the USA
Source: Sci Rep. 2025 Aug 5;15:28542. doi: 10.1038/s41598-025-12972-x (PMC12325701; doi:10.1038/s41598-025-12972-x)
Supplement: Supplementary file 1 — Supplementary Material 1 [file 41598_2025_12972_MOESM1_ESM.pdf]

Supplementary material:

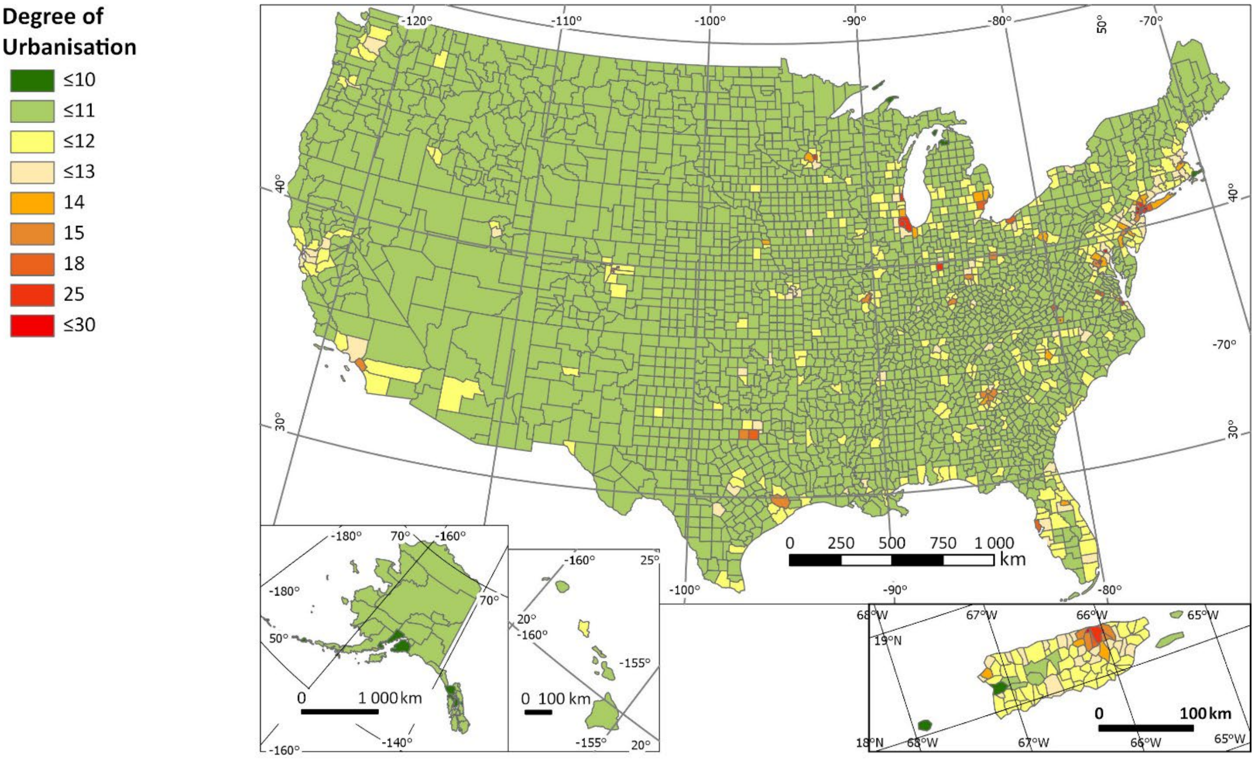

Calculated from GHSL - Global Human Settlement Layer
European Commission:
https://ghsl.jrc.ec.europa.eu/download.php?ds=smod

Map shows mean of Degree of Urbanisation for each county

Supplementary file 1: Degree of urbanization that was used to normalize the data (excl Puerto Rico).

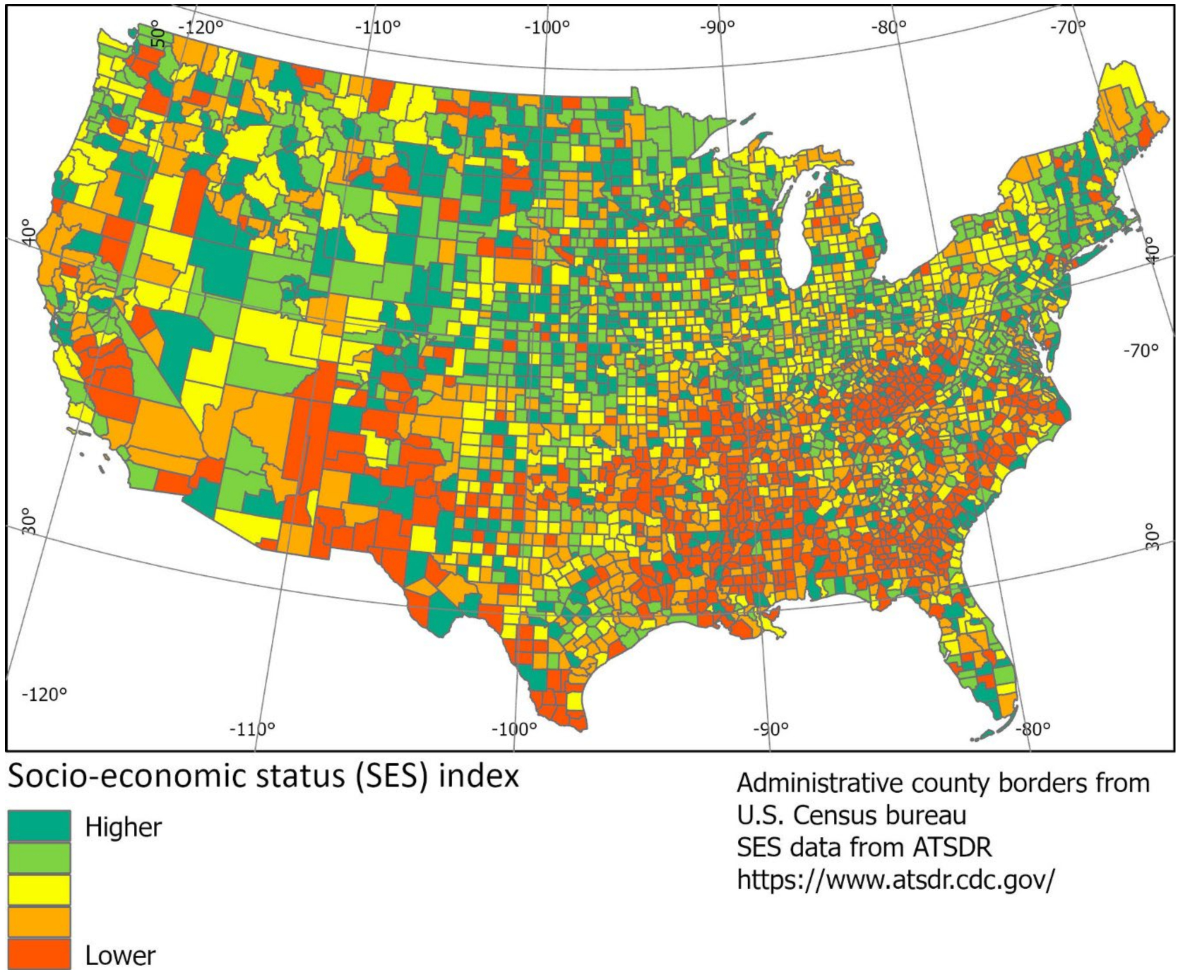

Supplementary file 2: Social-economic status (SES) index that was used to normalize our data (incl Alaska and Hawaii).

Supplementary file 3: Regression output from Fixed Effects regression

VARIABLES	COVID-19 Mortality Incidence
Urbanization index	
11	40.18***
	(18.45 - 61.91)
12	25.88*
	(-4.203 - 55.97)
13	26.36
	(-11.36 - 64.09)
14	29.33
	(-15.12 - 73.79)
15	41.92
	(-17.31 - 101.2)
16	27.00
	(-28.80 - 82.81)
17	31.34
	(-21.47 - 84.14)
18	59.07
	(-15.46 - 133.6)
19	78.75
	(-28.69 - 186.2)
20	51.17
	(-32.87 - 135.2)
21	-78.32
	(-227.5 - 70.83)
22	-8.225
	(-96.70 - 80.25)
23	16.67
	(-90.93 - 124.3)
24	37.18
	(-56.54 - 130.9)
25	31.00
	(-98.51 - 160.5)
26	-17.39
	(-166.5 - 131.7)
27	24.23
	(-105.5 - 154.0)
28	94.12
	(-88.16 - 276.4)
29	120.2**
	(25.33 - 215.1)
30	-6.680

	(-87.87 - 74.51)
Continentality Index	
4	-50.19
	(-190.4 - 90.06)
8	-10.60
	(-179.5 - 158.3)
9	-26.06
	(-293.5 - 241.4)
10	-10.65
	(-133.2 - 111.9)
12	-33.27
	(-137.0 - 70.42)
13	-12.99
	(-121.1 - 95.08)
14	19.25
	(-91.35 - 129.8)
15	39.29
	(-65.92 - 144.5)
16	91.09*
	(-11.30 - 193.5)
17	66.30
	(-25.08 - 157.7)
18	72.51*
	(-13.05 - 158.1)
19	105.8**
	(20.36 - 191.2)
20	112.9***
	(27.84 - 198.0)
21	87.79**
	(3.885 - 171.7)
22	77.72*
	(-6.375 - 161.8)
23	86.83**
	(3.154 - 170.5)
24	81.60*
	(-1.936 - 165.1)
25	99.07**
	(15.67 - 182.5)
26	124.7***
	(40.18 - 209.3)
27	133.6***
	(48.31 - 218.9)
28	136.3***

	(50.56 - 222.0)
29	97.69**
	(10.16 - 185.2)
30	97.96**
	(9.983 - 185.9)
31	114.5**
	(26.57 - 202.4)
32	124.6***
	(34.62 - 214.6)
33	126.0***
	(36.09 - 216.0)
34	162.5***
	(71.62 - 253.4)
35	189.7***
	(97.32 - 282.0)
36	138.1***
	(42.81 - 233.4)
37	148.7***
	(49.12 - 248.2)
38	182.8***
	(77.88 - 287.8)
39	-26.64
	(-295.1 - 241.8)
41	84.20
	(-183.6 - 352.0)
r_ses_nopr	239.4***
	(218.9 - 259.9)
Share of population over 65	8.736***
	(7.632 - 9.839)
Constant	-53.48
	(-144.1 - 37.12)
Observations	3,030
R-squared	0.258
Number of region_1	4

95% Confidence intervals in parentheses
*** p<0.01, ** p<0.05, * p<0.1

Supplementary file 3: Regression output from Fixed Effects regression

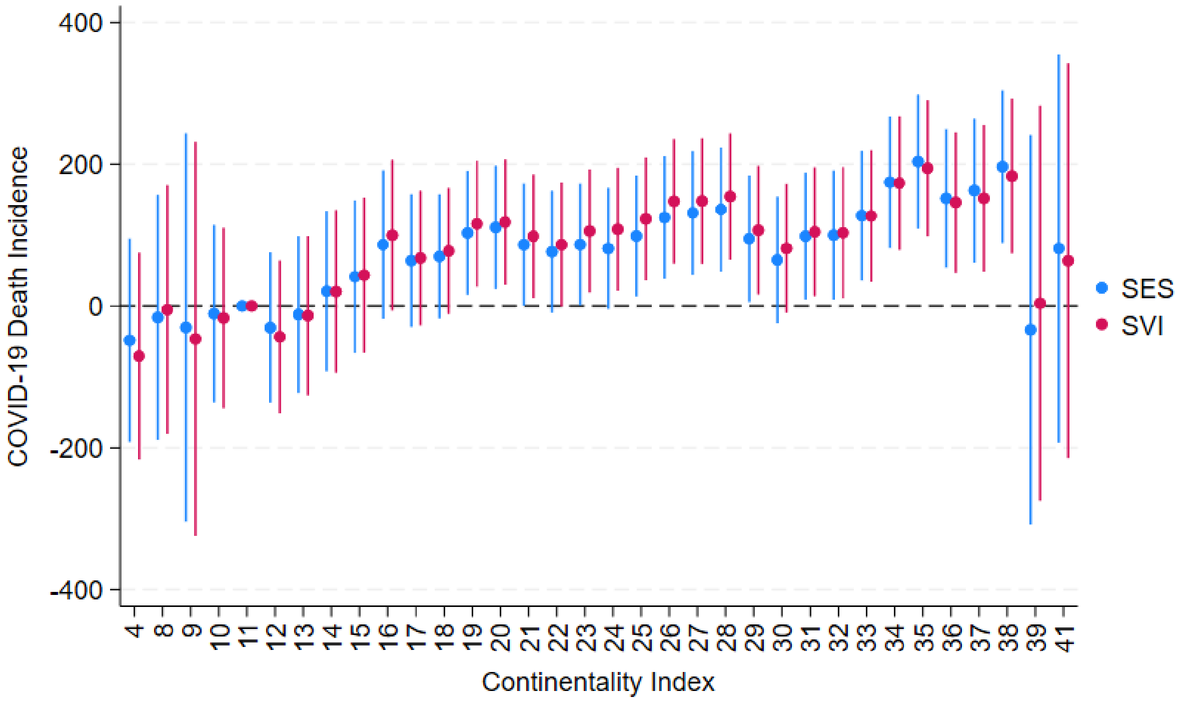

Supplementary file 4: The mortality/continentality coefficient, normalized with the factors we used (over 65 year olds, degree of urbanisation, SES) and a replacement of the SES for the entire SVI. The differences are not statistically significant.
